# Prognostic profile of systemic sclerosis: analysis of the clinical EUSTAR cohort in China

**DOI:** 10.1186/s13075-018-1735-4

**Published:** 2018-10-22

**Authors:** Shasha Hu, Yong Hou, Qian Wang, Mengtao Li, Dong Xu, Xiaofeng Zeng

**Affiliations:** Department of Rheumatology and Clinical Immunology, Peking Union Medical College Hospital, Chinese Academy of Medical Sciences & Peking Union Medical College, Beijing, 100730 China

**Keywords:** Systemic sclerosis, Prognosis, Cause of death

## Abstract

**Background:**

Systemic sclerosis is a disease that has significant clinical heterogeneity. This study aims to determine the causes and risk factors of death in a single center European League Against Rheumatism Scleroderma Trials and Research Group (EUSTAR) cohort at the Peking Union Medical College Hospital (PUMCH) in China.

**Methods:**

Patients clinically diagnosed with systemic sclerosis (SSc) between Feb 2009 and Dec 2015 were prospectively recruited from the EUSTAR database and Chinese Rheumatism Data Center (CRDC) of the PUMCH. Baseline and follow-up data were collected. Kaplan-Meier analysis was used to estimate survival, and Cox proportional hazards regression analysis was used to identify factors associated with mortality.

**Results:**

A total of 448 patients were included in the cohort, of whom 56.7% had limited cutaneous systemic sclerosis (lcSSc). The average age at diagnosis was 42.8 ± 12.1 years. The prevalence of interstitial lung disease (ILD) was 382/447 (85.5%). Among 402 patients, 348 of them took glucocorticoid during the disease course; 374 patients received immunosuppressors. Across 2167 patient-years, 40 patients died. Of these, 27 deaths were attributable to SSc, with pulmonary arterial hypertension (PAH) being the leading cause of death. The median survival time was 53 months. Survival rates from disease diagnosis were 97.0%, 94.6%, 91.1% and 87.8% at 1, 3, 5 and 10 years, respectively. Independent prognostic factors for mortality were PAH (HR 6.248, 95% CI 2.855, 13.674) and arrhythmia (HR 4.729, 95% CI 1.588, 14.082). Tripterygium wilfordii Hook F (TwHF) (log-rank test 7.851, *p* 0.005) and methotrexate (MTX) (log-rank test 7.925, *p* = 0.005) were found in survival analysis to be protective treatments against mortality. Patients who used cyclophosphamide (CTX) during the disease course had poorer prognosis (log-rank test 5.177, *p* = 0.023).

**Conclusions:**

In china, although there is a high prevalence of ILD in patients with SSc (85.5%), most of them have reserved pulmonary function, which means that interstitial lung disease (ILD) is not the most important factor in the death of patients with SSc and also is not a risk factor for poor prognosis. Only ILD with pulmonary dysfunction is associated with poor outcome. The 10-year cumulative rate (87.8%) in patients with SSc in China is slightly lower than the Europe, and pulmonary arterial hypertension (PAH) and arrhythmia at baseline are independent prognostic factors, whereas PAH instead of ILD is the leading cause of death in patients with SSc. Interestingly, the Chinese traditional medicine TwHF, as a protective factor for survival deserves further study.

**Electronic supplementary material:**

The online version of this article (10.1186/s13075-018-1735-4) contains supplementary material, which is available to authorized users.

## Background

Systemic sclerosis (SSc) is an autoimmune disease of unknown etiology that is characterized by microvasculopathy, immune system disturbances and increased tissue deposition of collagen in the skin and internal organs. The disease course is unpredictable and can remain relatively stable or rapidly progress. Several clinical cohort studies of SSc have been carried out in many countries to determine the clinical features and survival prognosis of patients with SSc [1, 2].

A number of studies suggest that SSc pathogenesis is influenced by multiple factors such as geography and ethnicity, whereas SSc prognosis is closely related to disease subtypes, antibody profile and visceral involvement [3]. Some studies have revealed that the incidence of SSc and diffuse cutaneous SSc (dcSSc) is higher in African-Americans who have worse disease prognosis [4]. A retrospective analysis compared clinical manifestations between Canadian patients of different descent and showed that those of Chinese descent have less severe disease, with less frequent gastrointestinal involvement and less severe vasculopathy than patients of European descent [5]. However, there have been limited clinical and prognostic large-scale clinical analyses of patients with SSc in China. A multicenter study conducted by Jiucun Wang [6], including 419 Chinese patients with SSc in Shanghai, Hebei Province, Sichuan Province and Hunan Province, showed that there are significant differences in the proportion of clinical subsets and frequencies of SSc-related autoantibodies compared to patients of US Caucasian descent. Another study recruited 1479 Taiwan patients based on the health insurance database and showed a lower incidence of SSc in Asian countries than in the USA or Europe [7]. However, neither of these studies had analyzed the clinical characteristics of patients with SSc in China.

Our previous study found that digital ulcers and telangiectasia were common in Chinese patients with SSc, with a prevalence of approximately 30% and 41.7%, respectively, which was similar to those reported abroad [8–11]. The differences in visceral involvement and autoantibody spectrum will determine the prognosis of Chinese patients with SSc. The aim of this study was to determine the prognosis, the cause of death and the risk factors for patients with SSc at a single Chinese center.

## Methods

### Patients

Patients clinically diagnosed with SSc between Feb 2009 and Dec 2015 were prospectively recruited from the European League Against Rheumatism (EULAR) Scleroderma Trials and Research Group (EUSTAR) and Chinese Rheumatism Data Center (CRDC) database of the Peking Union Medical College Hospital (PUMCH). Diagnosis of SSc was fulfilled according to the 2013 American College of Rheumatology (ACR)/EULAR criteria. Ethics committee approval was obtained for the EUSTAR and CRDC study and all subjects provided informed written consent.

### Data collection

Patients were mainly followed up by outpatient visits at intervals of 6 months to 1 year. Demographic, clinical and laboratory data were collected and entered into a database according to consolidated regulations. Patients whose last follow-up date was more than 1 year from May 2016 were followed up by telephone.

Patients were classified into limited and diffuse cutaneous subsets based on the definition of Leroy et al. [12]. Identification of peripheral vascular involvement included Raynaud’s phenomenon (RP), fingertip ulcer, loss of finger pads/pitting scars and telangiectasia. Identification of lung involvement included interstitial lung disease (ILD) and pulmonary arterial hypertension (PAH). ILD was defined as ground glass opacification or fibrosis on high-resolution computed tomography (HRCT). PAH was defined as a mean pulmonary arterial pressure > 25 mmHg at rest, together with pulmonary capillary wedge pressure < 15 mmHg determined by right heart catheterization or pulmonary artery systolic pressure (PASP) > 40 mmHg at rest based on an echocardiogram test. Those who only took echocardiogram test and hadn't taken right heart catheterization once in series and had maximum tricuspid regurgitant velocity (TRV) and pulmonary artery systolic pressure (PASP) of 2.9–3.4 m/s and 37–50 mmHg, respectively, were ruled out as diagnosis of PAH could not be confirmed [13]. Cardiac involvement included arrhythmia, left ventricular dysfunction (LVEF< 50%), decline of left ventricular diastolic function, pericardial effusion and valvular disease that could not otherwise be explained. Gastrointestinal involvement, according to the definition in the EUSTAR database, including esophageal (based on the presence of heartburn, regurgitation or dysphagia symptoms), gastric (based on clinical symptoms of early satiety, flatulence and vomiting) and intestinal involvement (diarrhea or constipation together with pseudo-obstruction secondary to small bowel involvement), in the absence of other explainable causes like treatment, cancer, etc.

Age at disease onset was defined as the age at first non-RP SSc manifestation, and age at disease diagnosis was defined as the age when SSc was diagnosed by a specialist. Length of follow up was defined as the time between the date of SSc diagnosis and last visit or death. Disease duration was defined as the time between the first non-RP SSc manifestation and last visit or death. Patients who were ≥ 60 years old at the time of disease onset were defined as having late-onset SSc.

Survival status was determined through the end of May 2016 based on database records or telephone tracing of patients for whom no data in the database had been entered for ≥ 12 months. The final status of loss to follow up was defined as having no data entered for ≥ 12 months with a failure to contact the patient on least two attempts. Data on cause of death were collected via medical records for patients who died in the PUMCH or for whom death certification was provided by relatives of patients who died in other hospitals. Deaths attributable to SSc were based on evaluation by three experienced rheumatologists from PUMCH.

### Statistical analysis

The data were analyzed using SPSS 19.0 and differences between groups were analyzed by analysis of variance (ANOVA), the Mann-Whitney test or the chi-square (χ^2^) test, depending on the distribution of the variables. Bivariate odds ratios with 95% confidence intervals (CI) were calculated. Kaplan-Meier analysis was used to estimate survival from the date of diagnosis, with the Mantel-Haenszel statistic (log-rank test) used to analyze differences in survival. Cox analysis and logistic regression analysis were used to obtain independent risk factors for SSc prognosis.

## Results

### Clinical and laboratory characteristics

A total of 448 patients with SSc were recruited for this study and 90.4% (405) were female. Limited cutaneous SSc (lcSSc) (254, 56.7%) was more common than diffused cutaneous SSc (dcSSc) (194, 43.3%). Overlap syndrome was present in 36 patients (8%). The mean age at disease onset and diagnosis was 39.0 ± 12.5 (8–75 years) and 42.8 ± 12.1 years, respectively. A significantly greater number of patients experienced disease onset at age< 60 years (427 vs. 21). Across 2167 patient-years, the median disease duration and length of follow up was 7.0 years (0.01, 49.4) and 53.5 months (0.25, 365), respectively. The majority of the cohort was Han Chinese, but 11 ethnic minorities were also included (4 Manchus, 5 Mongolian and 2 Korean). Past history of cardiovascular and respiratory diseases was as follows: 47 patients had a history of hypertension, 9 had previous coronary heart disease, 6 had previous cerebrovascular disease, 1 had rheumatic heart disease and 1 had silicosis.

Raynaud’s phenomenon was the most common peripheral vascular involvement in patients with SSc in this cohort (424, 94.6%), 72.3% of whom (315) presented with Raynaud’s phenomenon as the first disease manifestation at disease onset. More than half of the patients (271 patients, 60.5%) had different respiratory symptoms, with shortness of breath after exercise (250, 55.8%) being the most common, followed by cough (106, 23.7%) and dyspnea (38, 8.5%). Most (358 patients, 80.1%) were diagnosed as having SSc-ILD at baseline, whereas 55 had concurrent ILD and PAH.

Pulmonary function test (PFT) had been undertaken in 80.8% of patients (*n* = 362) at baseline, and the mean ± SD for forced expiratory volume in 1 s (FEV1), total lung capacity (TLC), forced vital capacity (FVC), diffusing capacity for carbon monoxide (DLCO)% on PFT were 82.1 ± 15.4, 7.8 ± 17.5, 81.2 ± 17.0, and 62.2 ± 20.0, respectively. Other organs involved at baseline are shown in Table 1. Elevated erythrocyte sedimentation rate (ESR) occurred in 121 patients (30.9%). C3 and C4 levels at baseline were 1.03 ± 0.25 g/L (in 314 cases) and 0.20 ± 0.09 g/L (in 312 cases), respectively. The average immunoglobulin G (IgG) level was 15 mg/dl (6.5, 52.2). Antinuclear antibodies (ANA) were seen in 97.7% of patients (336/344), whereas the presence of anti-ribonucleoprotein (RNP) antibody was less frequent (90/344, 26.2%). Anti Scl-70 and anticentromere antibody (ACA) were present in 46.8% (169/361) and 16.5% (48/291), respectively, of patients, 3 of whom were double positive for anti Scl-70 and ACA.

**Table 1:** Clinical features at baseline and during follow up

	Total	Incident
	*N* = 448	*N* = 448
	Baseline, number (%)	Follow up, number
Vascular involvement	430 (96.0)	10
Raynaud phenomenon	424 (94.6)	11
Fingertip ulcer	128 (28.6)	19
Loss of finger pad	137 (30.6)	20
Telangiectasia	153 (34.2)	39
Arthritis	121 (27.0)	66
Muscle involvement	44 (9.8)	11
Pulmonary interstitial disease (ILD)^a^	358/447 (80.1)	24
Pulmonary arterial hypertension (PAH)	67/359 (18.7)	11
Gastrointestinal involvement	274 (61.2)	38
Esophagus	243 (54.2)	40
Gastric	115 (25.7)	20
Intestinal	67 (15.0)	13
Cardiac involvement	177/397 (44.6)	97/411
Arrhythmia	16/396 (4.0)	13/412
LVEF < 50%	1/396 (0.3)	3/411
Left ventricular diastolic dysfunction	69/396 (17.4)	70/411
Pericardial effusion	57/396 (14.4)	53/411
Valvular disease	88/396 (22.2)	70/412
Renal crisis	5 (1.1)	0
Skin Rodnan score	6 (0, 43)	–

*LVEF* left ventricular ejection fraction

^a^Pulmonary interstitial disease: one patient diagnosed with silicosis at first visit was excluded

During follow up (53 months (0.25, 365)), 24 patients developed pulmonary interstitial diseases, and the prevalence of ILD reached 85.5%. PAH was identified in 78/361 patients, accounting for 21.6% of the cohort, and 63 patients had both PAH and ILD. Arrhythmia was present in 29 patients: among those with arrhythmia, only 5 patients experienced palpitations and other symptoms. At baseline, one patient had ovarian cancer and one had thyroid carcinoma, whereas two patients developed tumors during the follow-up period, including one with lung adenocarcinoma and one with leukemia.

Among 402 patients, 348 of them took glucocorticoid during the disease course: 374 patients received immunosuppressors and 43 of them took only immunosuppressors; 11 patients took neither glucocorticoid nor immunosuppressors. Among patients who used immunomodulators during follow up, 250 patients took cyclophosphamide (CTX), 2 took cyclosporin (CsA), 28 took mycophenolate mofetil (MMF), 5 took tacrolimus, 2 took azathioprine (AZA), 9 took leflunomide (LEF), 103 took TwHF, 108 took MTX and 29 took hydroxychloroquine (HCQ); 102 patients used two immunomodulators at the same time and 19 used three immunomodulators at the same time.

### Survival analysis

**Fig. 1:**
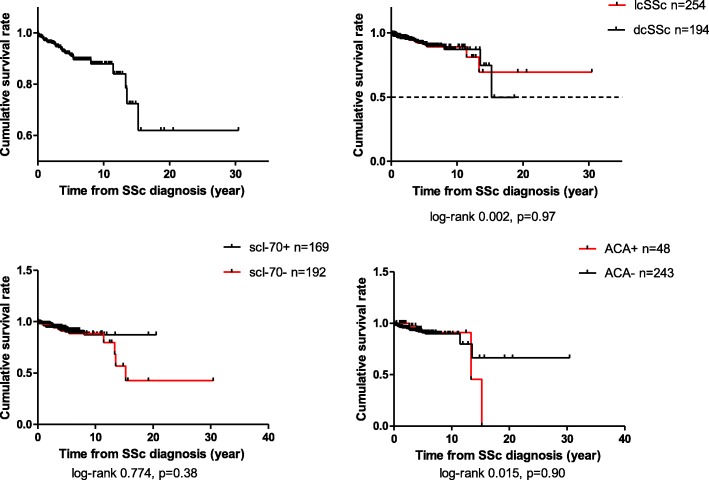
Survival curve and subgroup study of patients with systemic sclerosis (SSc) in the Peking Union Medical College Hospital (PUMCH) cohort. lcSSc, limited cutaneous SSc; dcSSc, diffuse cutaneous SSc; ACA, anticentromere antibody

The 448 patients with SSc were followed up for a total of 2167 patient-years. The median survival time for the patient cohort was 53 months (0.25, 365). Survival rates from disease diagnosis were 97.0%, 94.6%, 91.1% and 87.8% at 1, 3, 5 and 10 years, respectively (Fig. 1). The cumulative survival rates at 1, 3, 5 and 10 years in patients with lcSSc were 97.6%, 94.2%, 91.1% and 89.1%, respectively, whereas for patients with dcSSc the rates were 96.3%, 95.1%, 91.2% and 87.0%, respectively.

No significant differences were seen in subgroup survival analysis according to subtypes (log-rank test 0.002, *p* = 0.968), baseline vascular (log-rank test 2.407, *p* = 0.121), joint (log-rank test 2.868, *p* = 0.090), muscular (log-rank test 1.009, *p* = 0.315), gastrointestinal (GI) involvement (log-rank test 0.027, *p* = 0.870), gender (log-rank test 0.014, *p* = 0.907) and age of disease onset (log-rank test 0.249, *p* = 0.618) (Additional file 1: Figure S2).

**Fig. 2:**
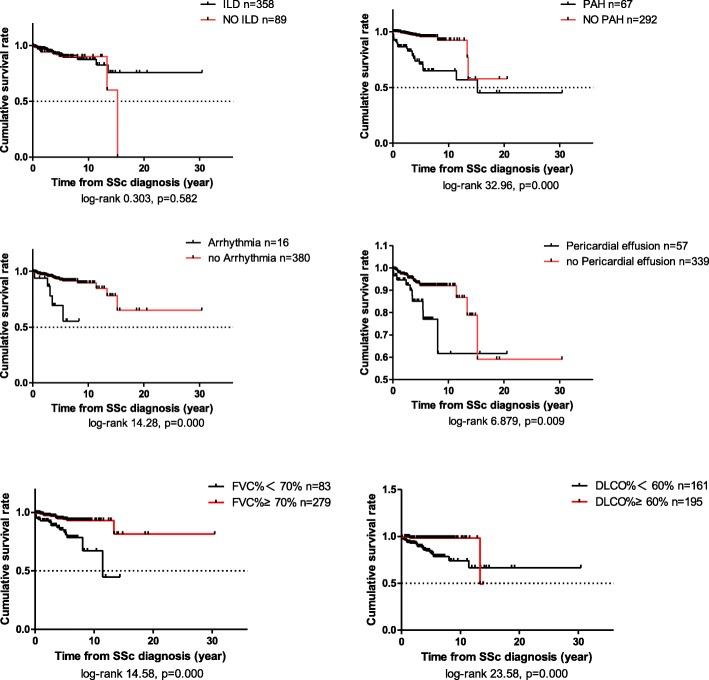
Survival analysis based on interstitial lung disease (ILD), pulmonary arterial hypertension (PAH), cardiac involvement and pulmonary dysfunction. SSc, systemic sclerosis; FVC, forced vital capacity; DLCO, diffusing capacity for carbon monoxide

The median modified Rodnan skin score (mRSS) was 6 points (0, 43) in this cohort. According to the distribution of skin scores, 87.5% of patients had a skin score < 16 points. Patients with higher skin score (mRSS > 15) had a poorer prognosis (log-rank test 7.977, *p* = 0.005). However, survival was better in patients who had no respiratory symptoms (Additional file 1: Figure S1) and less pulmonary dysfunction (FVC% < 70%, log-rank test 14.58, *p* = 0.000, DLCO% < 60%, log-rank test 23.58, *p* = 0.000) (Fig. 2).

No significant differences were seen in subgroup survival analysis according to use of HCQ (log-rank test 1.186, *p* = 0.276) or MMF (log-rank test 0.016, *p* = 0.9). TwHF (log-rank test 7.851, *p* = 0.005) and MTX (log-rank test 7.925, *p* = 0.005) were found to be protective against mortality in survival analysis. Patients who took CTX during the disease course had a poorer prognosis (log-rank test 5.177, *p* = 0.023).

Patients who had PAH (log-rank test 32.96, *p* = 0.000), arrhythmia (log-rank test 14.28, *p* = 0.000) and pericardial effusion (log-rank test 6.879, *p* = 0.009) at baseline had a poorer prognosis than those who did not. However, there was no significant difference between patients with or without ILD at baseline (log-rank test 0.303, *p* = 0.582). Among 288 patients who had both HRCT-confirmed ILD and PFT simultaneously, those who had pulmonary dysfunction (defined as FVC < 70% or DLCO < 60%) had a poorer prognosis (log-rank test 16.266, *p* = 0.000) (Fig. 2).

Among 358 patients who had ILD at baseline, 217 of them had continuous monitoring by HRCT. We qualitatively assess disease progression according to the description in the radiology reports: 64 patients had radiographic evidence of aggravation (median disease duration at baseline was 0 (0, 132) months) during the disease course, and 153 patients remained stable or improved (median disease duration at baseline was 1 (0, 165) month). A poor prognosis was not confirmed among patients with ILD who had radiographic aggravation (log-rank test 2.629, *p* = 0.105). There were 89 patients who did not have ILD at baseline, and 24 of them developed ILD during the disease course. New onset of ILD was not associated with a poor prognosis (log-rank test 0.111, *p* = 0.740).

Patients with scleroderma renal crisis (SRC) were rare in this cohort. Despite the rarity, patients with SRC at baseline had a poor outcome compared to those patients that did not have SRC (log-rank test 11.36, *p* = 0.001).

### Risk factors for prognosis

Univariate survival analysis suggested that SRC, PAH, arrhythmia, pericardial effusion, skin score > 15, FVC% < 70% and DLCO% < 60% on pulmonary function tests (PFT), and respiratory symptoms at baseline were associated with poor prognosis. Further Cox multivariate analysis confirmed that PAH (hazard ratio (HR) 6.248, 95% CI 2.855, 13.674) and arrhythmia (HR 4.729, 95% CI 1.588, 14.082) at baseline are independent risk factors for poor outcome in patients with SSc.

### Cause of death

Among the cohort, 40 patients with SSc died (Table 2): 27 deaths (67.5%) were related to SSc. PAH was the leading cause of death, accounting for 55% of all deaths. The mortality rate in patients who had PAH during the disease course was 28.2% (22/78 patients). Of the 63 patients in this cohort who had both PAH and ILD, 17 died (mortality rate, 27.0%). Among the four patients who died from myocardial infarction and stroke, only one had no history of cardiovascular involvement or hypertension. The two patients who died of lung infection both had SSc-related lung involvement, one had PAH, and the other had ILD. One patient died of bacteremia due to cholecystitis. Two patients who died had tumors and one died of leukemia 5 years after the SSc diagnosis. The other patient was first treated for hemoptysis and within one year of SSc diagnosis this patient died from lung adenocarcinoma that had metastasized to the bone, meninges and liver.

**Table 2:** Causes of death in the patient cohort

Cause of death	Deaths, *N* = 40 (%)
All causes of death	40
SSc-related deaths	27 (67.5)
Lungs	23 (57.5)
ILD	1
PAH	22
Heart	1 (2.5)
Arrhythmia	–
Heart failure	1
Renal crisis	3 (7.5)
Non-SSc-related deaths	10 (25.0)
Infection	3 (7.5)
Pulmonary infection	2
Bacteremia	1
Malignant tumor	2 (5.0)
Adenocarcinoma of lung	1
Leukemia	1
CCVd	4 (10.0)
Mi	1
Cerebral apoplexy	3
Digestive tract bleeding	1 (2.5)
Unknown cause of death	3 (7.5)

Results are presented as number (percentage) of patients

*ILD* interstitial lung disease, *PAH* pulmonary arterial hypertension, *SSc* systemic sclerosis, *CCVd* chronic cerebrovascular disease, *Mi* myocardial infarction

## Discussion

Systemic sclerosis is a disease that has significant clinical heterogeneity. Some patients progress rapidly and even die, whereas others remain stable and have limited symptoms such as finger sclerosis and minimal visceral involvement. Therefore, treatment strategies for SSc would benefit from the ability to predict prognosis based on clinical manifestations or laboratory parameters in the early stage of the disease.

Earlier studies reported a 10-year survival rate for SSc that was as low as 50% [14], whereas more recent studies including the EUSTAR registry reported 5-year and 10-year survival rates of 90% and 84%, respectively [15] (Clinical manifestation and survival rates published for different countries since 2008 are listed in (Additional file 2) [16–23]). In recent years, the SSc survival rate has improved. Indeed, Ferri et al. analyzed prognostic cohorts of SSc worldwide at different times and found that the median values of cumulative 10th-year survival rates in patients with SSc increased from 54% to 83.5% [24]. Unfortunately, we could not make comparisons with the EUSTAR study, because no specific organ involvement was mentioned. Compared with two contemporary European studies and one Asian study, the incidence of ILD and PAH in Chinese patients with SSc were much higher than those in Spain, which might partially explain the reduction in 3-year and 5-year survival rates. Compared with the Italian study in 2010, despite a higher incidence rate of PAH in Chinese patients, the 3-year survival rate was similar. The 5-year survival rate in Italy was lower than in China, which might be related to the significant increase in SRC and GI involvement; the latter may result in malnutrition and susceptibility to infection. Although having a high incidence of ILD and PAH, as in China, Thailand had a slightly lower survival rate due to there being a greater proportion of patients with dcSSc in the cohort. Therefore, it can be seen that the characteristics of organ involvement in patients with SSc in different countries determine diverse prognoses.

Survival among patients with dcSSc in our cohort was consistent with that in a Spanish report [2], but was higher than that reported in other studies conducted during the same period [21]. The mortality rate can be underestimated due to the failure to capture data on early death in patients with dcSSc, which could result in overestimation of survival in these patients [25]. Moreover, patients with dcSSc were reported to have worse outcomes [16, 17, 19, 20] relative to patients with lcSSc, due to their predisposition toward internal organ involvement, specifically lung and renal involvement. Our study confirmed that a higher mRSS, which manifests as more extensive skin involvement, was a risk factor for mortality in patients with SSc, but no significant difference in 1, 3, 5 and 10-year survival rates was seen between disease subtypes.

In this study, we found that vital organ involvement such as renal crisis, PAH, cardiac involvement (especially arrhythmia and pericardial effusion), severe skin involvement, presence of respiratory-related symptoms and decreased FVC% and DLCO% on PFT were risk factors for poor prognosis. Multivariate Cox analysis confirmed that PAH and arrhythmia at baseline were independent prognostic factors in this cohort. Arrhythmia can have several origins that are related to primary heart involvement such as cardiac conductive tissue fibrosis and myocardial fibrosis [26, 27], pericardial disease or PAH [28], and was associated with poor outcome [15]. In this study, confirming a diagnosis of arrhythmia during the early disease stage was challenging, as the majority of patients had no clinical symptoms or signs on standard electrocardiogram (ECG) at rest [28].

Although ILD was an independent prognostic factor in a previous study [4], our study did not confirm this, which might be due to the large proportion of patients with ILD and the inclusion of patients with more early or mild interstitial lesions in our study due to the reliance on HRCT imaging features to define ILD. Our study did not confirm a poor prognosis among patients with ILD who had radiographic evidence of aggravation, partially due to individual differences among radiologists in the assessment of images and lack of quantitative assessment. Our findings suggest that ILD with pulmonary dysfunction (FVC < 70% or DLCO < 60%), rather than the presence of ILD alone, is associated with poor prognosis.

Patients who took CTX during the disease course had poorer prognosis. It was probably because patients requiring CTX were more prone to significant organ involvement and they could have been affected by combined medication and side effects. TwHF, a traditional Chinese herbal medicine, had been widely used in autoimmune diseases, including rheumatoid arthritis (RA), systemic lupus erythematosus (SLE) and SSc etc., mainly due to their anti-inflammatory immunoregulation and favorable cost-benefit ratio. Our previous study showed that TwHF treatment for more than 1 year could improve FVC (0.11 ± 0.25) and FVC% (3.83 ± 8.58%) (*p* < 0.05) (not published). The protective effect of TwHF on survival in patients with SSc deserves further study.

The median follow-up time of the 40 patients who died was 31.5 months, but the average follow-up time of the survivors was 56 months, indicating that there was no bias associated with a longer observation period among the patients with SSc who died.

A meta-analysis of death in patients with SSc showed that heart and lung involvement had replaced renal crisis as the leading cause of death in patients with SSc [29]. SSc-related deaths, especially lung involvement, were the leading cause of death in patients with SSc in this study, accounting for 67.5% of deaths, which was similar to results for a multinational inception cohort (patients recruited within 4 years of disease onset, 62.1%) [25]. The death rate attributable to lung involvement in patients with SSc increased over time [30]. In the EULAR cohort, 19% of the patients died of ILD, which was higher than that for PAH (14%), but in the study cohort here, only one patient died of ILD, and thus PAH was the leading cause of death, consistent with some other studies [16, 25, 31]. This outcome might also be related to coexisting ILD in 17 out of 22 patients with SSc who died of PAH. To determine the cause of non-sudden cardiac death in patients with both PAH and ILD can be indistinguishable, as the accuracy of the physician’s judgment might affect the result and underestimate deaths due to ILD. Only one patient died of cardiac involvement among the SSc related deaths, and this small number was related to the small number cases of patients who had severe cardiac involvement.

With the progress of disease screening and treatment, patients with SSc can have longer life expectancy, and thus long-term complications could become particularly important later in the disease course. Cardiovascular and cerebrovascular disease, infections and tumors were the main causes of non-SSc related deaths in this cohort, as was also seen in a study by Hao et al. [25]. A number of reports confirmed that the incidence of cancer SSc in patients was elevated [32]. The incidence of malignant tumors in patients with SSc is 3–11%, which was 1.5–5 times [33] higher than that for the healthy population. In this cohort, the two patients with malignant tumors during follow up were female, had the lcSSc subtype and were positive for anti scl-70 antibody. Patients with SSc had fewer hematologic malignancies compared with solid tumors, with only a few case reports of hematologic tumor such as multiple myeloma, chronic lymphocytic and myeloid leukemia (CLL and CML), Hodgkin’s and non-Hodgkin’s lymphoma and hairy cell leukemia [34].

There is no consensus about the incidence of coronary heart and cerebrovascular disease among patients with SSc compared with the general population. A study from the Australian Scleroderma Cohort Study (ASCS) reported that after adjusting for age, sex and traditional risk factors for atherosclerosis, patients with SSc were 3.2 times more likely to have coronary heart disease than the general population [19], whereas studies by Nordin et al. [35] and Hettema et al. [36] concluded that atherosclerosis was not more prevalent in patients with SSc than in controls.

## Conclusion

In china, although there is a high prevalence of ILD in patients with SSc (85.5%), most patients have reserved pulmonary function, which means that ILD is not the most important factor in the death of patients with SSc and is also not a risk factor for poor prognosis. Only ILD with pulmonary dysfunction is associated with poor outcome. The 10-year cumulative rate (87.8%) in patients with SSc in China is slightly lower than in Europe, and PAH and arrhythmia at baseline are independent prognostic factors, whereas PAH instead of ILD is the leading cause of death in patients with SSc. Interestingly, the Chinese traditional medicine TwHF deserves further study as a protective factor in survival. This is the first study of prognosis in Chinese patients with SSc, and a study involving multiple centers in China is in progress. The results from this and future studies should help elucidate the influence of various ethnic differences on SSc disease phenotype and prognosis.

## Additional files

Additional file 1: **Figure S1.** Survival analysis based on respiratory symptoms. **Figure S2.** Survival analysis based on organ involvement. (DOCX 550 kb)

Additional file 2: Clinical manifestation and survival rates published by different countries since 2008. (DOCX 19 kb)

## Abbreviations

ACA: Anticentromere antibody
ACR: American College of Rheumatology
ANA: Antinuclear antibody
ANOVA: Analysis of variability
ASCS: Australian Scleroderma Cohort Study
CCVd: Chronic cerebrovascular disease
CI: Confidence intervals
CLL: Chronic lymphocytic
CML: Chronic myeloid leukemia
CRDC: Chinese Rheumatism Data Center
CTX: Cyclophosphamide
dcSSc: Diffuse cutaneous systemic sclerosis
DLCO: Diffusing lung capacity for carbon monoxide
ESR: Erythrocyte sedimentation rate
EUSTAR: European League Against Rheumatism Scleroderma Trials and Research Group
FEV1: Forced expiratory volume at 1 min
FVC: Forced vital capacity
GI: Gastrointestinal
HRCT: High-resolution computed tomography
IgG: Immunoglobulin G
ILD: Interstitial lung disease
lcSSc: Limited cutaneous systemic sclerosis
LVEF: Left ventricular ejection fraction
Mi: Myocardial infarction
mRSS: Modified Rodnan skin score
MTX: Methotrexate
PAH: Pulmonary arterial hypertension
PASP: Pulmonary artery systolic pressure
PFT: Pulmonary function test
PUMCH: Peking Union Medical College Hospital
SRC: Scleroderma renal crisis
SSc: Systemic sclerosis
TRV: Tricuspid regurgitant velocity
TwHF: Tripterygium wilfordii Hook F
