# Supplementary material for: Prognostic profile of systemic sclerosis: analysis of the clinical EUSTAR cohort in China
Source: Arthritis Res Ther. 2018 Oct 22;20:235. doi: 10.1186/s13075-018-1735-4 (PMC6235213; doi:10.1186/s13075-018-1735-4)
Supplement: Supplementary file 1 — Figure S1. Survival analysis based on respiratory symptoms. Figure S2. Survival analysis based on organ involvement. (DOCX 550 kb) [file 13075_2018_1735_MOESM1_ESM.docx]

**Figure S1** **Survival analysis based on respiratory symptoms**

**Figure S2 Survival analysis based on organ involvement**
