# Supplementary material for: Prognostic profile of systemic sclerosis: analysis of the clinical EUSTAR cohort in China
Source: Arthritis Res Ther. 2018 Oct 22;20:235. doi: 10.1186/s13075-018-1735-4 (PMC6235213; doi:10.1186/s13075-018-1735-4)
Supplement: Supplementary file 2 — Clinical manifestation and survival rates published by different countries since 2008. (DOCX 19 kb) [file 13075_2018_1735_MOESM2_ESM.docx]

**Additional file 2. Clinical manifestation and survival rates published by different countries since 2008**

| Author, year (ref.) | Country | Recruitment period | No. of patients | 5-yr / 10-yr survival from disease diagnosis (%) | Subsets | Organ involvement (%) | Laboratory test (%) |
| --- | --- | --- | --- | --- | --- | --- | --- |
| Joven, 2010 [16] | Spain | 1980-2006 | 204 | (Calculated from disease onset) 85/75 | lcSSc/dcSSc/overlap  59%/31%/10% | Cardiac involvement: 82/204 (40)  PAH: 59/204 (29)  Pericardial effusion: 27/204 (13)  ILD： 78/203 (38)  Renal (SRC): 6/203 (3)  Gastrointestinal involvement: 114/203 (56) | ANA+ 187/201 (93)  ACA+ 75/200 (38)  Anti-Scl70+ 46/200 (23)  Elevated ESR 50/198 (25) |
| Al-Dhaher, 2010 [1] | Canada | 1994-2004 | 185 | 82/90 | lcSSc/dcSSc  63%/37% | Cardiac involvement: 53/185 (29)  PAH: 19 (14)  ILD: 27 (15)  SRC: 13 (7)  GI: 168 (91) | ANA+ 107 (59) |
| Vettori, 2010 [17] | Italy | 2000-2008 | 251 | 94.8/77.1 | lcSSc/dcSSc  79.7%/20.3% | mRSS: 5.6±6.7, 3 (0-36)  Cardiac involvement: 36 (14.3)  PAH: 7/185 (3.8)  ILD: 168 (66.9)  Renal*: 42 (16.7)  Including arterial hypertension, recent rise in Cr or preexisting SRC  GI: 188 (74.9) | ANA+ 61/246 (24.8)  ACA+ 95/246 (38.6)  Anti-Scl-70+ 90/246 (36.6) |
| Tyndall, 2010 [15] | EUSTAR | 2004.6-2008.4 | 5860 | 90/84 | Not mentioned | Not mentioned | Not mentioned |
| Walker, 2007 [18] |  | 2004.6-2006.4 | 3656 | - | lcSSc/dcSSc/overlap  36.9%/57.7%/5.6% | Unable to get original data | ANA+ 3346  Anti-Scl-70+ 1330  ACA+ 1106 |
| Hashimoto, 2001 [19] | Japan | 1973-2008 | 405 | (Calculated from disease onset) -/88 | lcSSc/dcSSc  32.6%/67.4% | Cardiac involvement: 79  PAH: 65  ILD:204  SRC:13  GI 187 | Anti-Scl-70+ 23.3%  ACA+ 36.1% |
| C-F Kuo, 2013[7] | Taiwan | 2002-2007 | 1479 (incident cases) | 83.2/- | Not mentioned | Not mentioned | Not mentioned |
| Sampaio, 2012 [20] | Brazil | 1991-2010 | 947 | 90/84 | lcSSc/dcSSc  75.2%/24.8% | mRSS＜20 635 (67.1) | ANA+ 839 (88.6)  Anti-Scl-70+ 152 (16.1)  ACA+ 209 (22.1) |
|  |  |  |  |  |  | PAH*: 221 (23.3)  *not associated to pulmonary fibrosis  ILD: 538 (56.8)  ILD+PAH: 132 (13.9)  SRC: 25 (2.6)  Esophageal (hypomotility or/and GERD) 897 (94.7) |  |
| Hoffmannvold, 2013 [21] | Norway | 1999-2009 | 312 | 91/70 | Unable to get original data | Unable to get original data | Unable to get original data |
| C.P, 2015 [2] | Spain | 2006-2008.1 | 879 | (Calculated from disease onset) 96/93 | lcSSc/dcSSc/ssSSc  64.5%/27.6%/7.8% | Cardiac involvement: 290 (33.0)  PAH: 161 (18.3)  ILD: 421 (47.9)  SRC: 24 (2.7) | ACA+ 356 (44.1) |
|  |  |  |  |  |  | GI: 613 (69.7) |  |
| Poormoghim, 2016 [22] | Italy | 1998.1-2012.8 | 220 | 92.6/82.3 | lcSSc/dcSSc  60%/40% | Skin score＜20/≥20 85%/15%  Cardiac involvement: 24.4%  PH (elevated PAP in ECHO): 16.2%  ILD (HRCT): 62.2%  SRC: 1.9% | ANA+ 91%  Anti-Scl-70+ 70.2%  ACA+ 8.4% |
| Wangkauw, 2017 [23] | Thailand | 2010.1-2014.8 | 115 (Early SSc pts) | Survival for 1, 2, 3 and 4 year  93, 91, 88, 88 | lcSSc/dcSSc  20.9%/79.1% | mRSS: 18.6±10.3 (for survivors)  31.3±13.6 (for deceased)  Cardiac involvement:  PAH: 4.3%  ILD: 73.0% | ACA+ 7.8% |
| Hu, 2018 | China | 2009.2-2015.12 | 448 | 91.1/87.8 | lcSSc/dcSSc  56.7%/43.3% | mRSS 6 (0, 43) | ANA+ 97.7%  Anti-Scl-70+ 46.8%  ACA+ 16.5% |
|  |  |  |  |  |  | Cardiac involvement: 44.6%  PAH: 21.6%  ILD: 85.5%  SRC: 1.1%  GI: 61.2% |  |
